# Application of single-cell sequencing technologies in pancreatic cancer

**DOI:** 10.1007/s11010-021-04095-4

**Published:** 2021-02-18

**Authors:** Mastan Mannarapu, Begum Dariya, Obul Reddy Bandapalli

**Affiliations:** 1 Department of Biotechnology, Dravidian University, Kuppam, Chittoor, Andra Pradesh 517 426 India; 2 Department of Bioscience and Biotechnology, Banasthali University, Vanasthali, Rajasthan 304022 India; 3 Medical Faculty Heidelberg, Heidelberg University, Heidelberg, Germany; 4 Hopp Children’s Cancer Center (KiTZ), Heidelberg, Germany; 5 Division of Pediatric Neurooncology, German Cancer Research Center, Heidelberg, Germany

**Keywords:** Pancreatic cancer, Single-cell sequencing, Intratumor heterogeneity, Circulating tumor cells, Metastasis, Transcriptome

## Abstract

Pancreatic cancer (PC) is the third lethal disease for cancer-related mortalities globally. This is mainly because of the aggressive nature and heterogeneity of the disease that is diagnosed only in their advanced stages. Thus, it is challenging for researchers and clinicians to study the molecular mechanism involved in the development of this aggressive disease. The single-cell sequencing technology enables researchers to study each and every individual cell in a single tumor. It can be used to detect genome, transcriptome, and multi-omics of single cells. The current single-cell sequencing technology is now becoming an important tool for the biological analysis of cells, to find evolutionary relationship between multiple cells and unmask the heterogeneity present in the tumor cells. Moreover, its sensitivity nature is found progressive enabling to detect rare cancer cells, circulating tumor cells, metastatic cells, and analyze the intratumor heterogeneity. Furthermore, these single-cell sequencing technologies also promoted personalized treatment strategies and next-generation sequencing to predict the disease. In this review, we have focused on the applications of single-cell sequencing technology in identifying cancer-associated cells like cancer-associated fibroblast via detecting circulating tumor cells. We also included advanced technologies involved in single-cell sequencing and their advantages. The future research indeed brings the single-cell sequencing into the clinical arena and thus could be beneficial for diagnosis and therapy of PC patients.

## Introduction

Pancreatic cancer (PC) is the third leading cause for cancer-related mortalities. As estimated by the American cancer society, 57,600 people were diagnosed with 47,050 deaths recorded due to this lethal disease for the year 2019 [1]. PC is the most common cancer occurring in both men and women worldwide. A tumor could be a distinct unit of collective cells and analysis of these cells would indeed provide an insight into the mechanisms underlying cancer progression and therapy resistance. As these cancer cells showed up with varied genetic mutations and gene expression, they vary in their morphologies, gene expression, progression rate, and metastatic potentiality [2]. Thus, this cellular heterogeneity caused due to varied environmental and genetic alterations is the major obstacle at present that is complicating the prognosis of cancer therapy. Traditionally available techniques for molecular profiling evaluate bulk sequencing to detect the mutations caused and dysregulated gene expression associated with cancer. However, identifying the rare clonal subpopulation is often a major task for researchers now [2]. The current single-cell technologies are the new avenues now, as they analyze mutation in DNA and gene expression in many distinct cells and indeed can potentially detect the resistant cell during or prior to the therapy [3]. This technique characterizes heterogeneity within and in between tumor, can rationalize mutation rate, and detect rare cell types eventually supporting the development of diagnostic and therapeutic guidelines.

The single-cell technology can be differentiated into 2 areas: single-cell separation and single-cell analysis. Wherein both the processes are interlinked and include laser capture microdissection (LCM), flow cytometry analysis (FCA), and microfluidics techniques for analysis. The cell sequence analysis includes genome (sequencing of DNA), epigenome (epigenetic markers), transcriptome (gene expression), and proteome (protein expression) [4]. These techniques provide an insight into cancer distinct metastasis and recurrence, among which genome analysis is found to be more encouraging for researchers and clinicians. Additionally, the single-cell technology efficiently detects rare tumor cells including cancer stem cells (CSCs), circulating tumor cells (CTCs), DNA methylation, distant metastasis, and intratumor heterogeneity (ITH) that further guides for individualized therapeutic strategies [5]. In this review, we have delineated application of single-cell technologies for PC. This article would extemporize a clear understanding of biological characteristics of the cancer progression and potentiate the diagnostic and therapeutic strategies for patients.

## Application of single-cell technology in detecting PC cells

### Analyzing intratumor heterogeneity

**Fig. 1:**
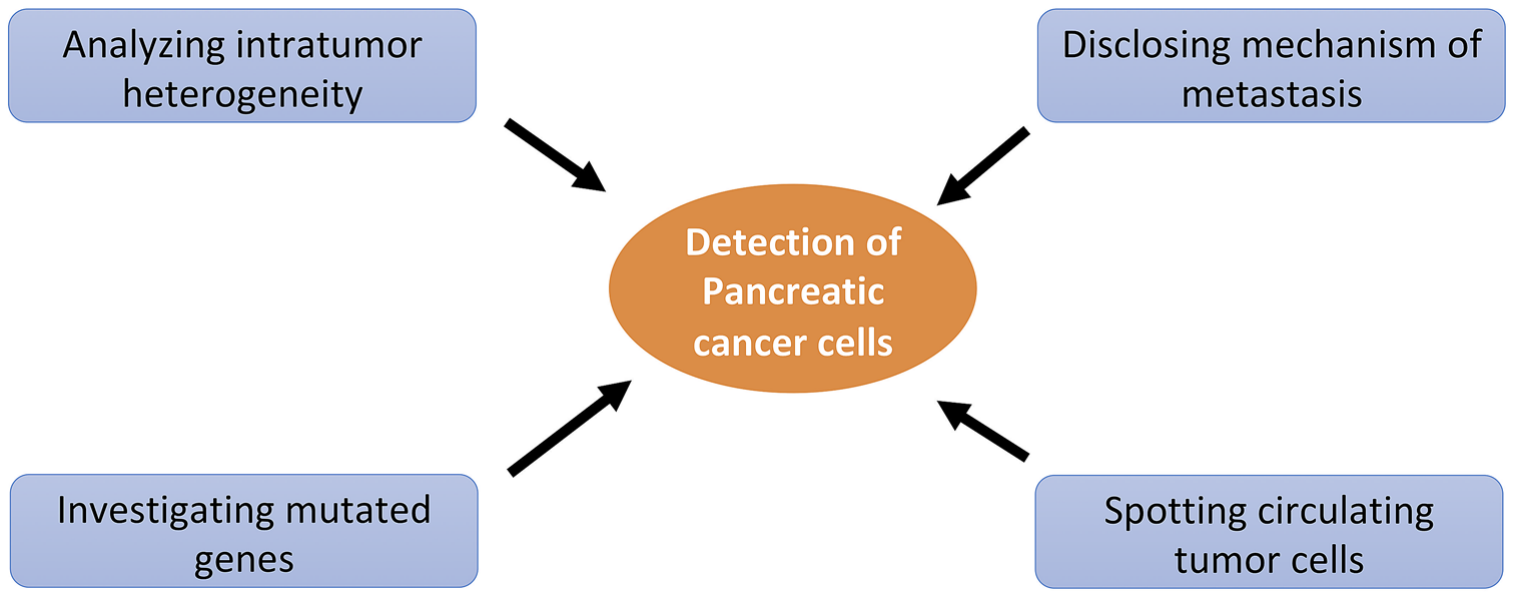
Applications of single-cell sequencing technology in detecting pancreatic cancer cell

PC is uniformly an aggressive disease with varied clinical heterogeneity that includes a complex of fibroblasts, and endothelial and inflammatory cells. These tumor cells also include a subpopulation of varied clonal cells in a single PC lesion that determines its complexity. Furthermore, the heterogeneity exists among patients of different stages that respond variedly for radio or chemotherapy. For instance, stage 3 and 4 PC patients having MSI high tumors respond for the first-line chemotherapy objectively that received immunotherapy and PARP inhibitors following platinum-based induction [6, 7]. They are found to be with stable disease and delay in disease progression, however, few patients do not respond to the treatment at all. Similarly, the heterogeneity is also observed in the resected patients that develop metastasis regardless of the traditional adjuvant therapies. Thus, a clear understanding of ITH promotes better prognosis and efficient disease prognosis with an effective therapy. Single-cell sequencing technology offers varied tools required for ITH in solid tumor cells like PC (Fig. 1). The development of powerful tools like ‘in deep analysis’ supports to get clear molecular resolution of tumor heterogeneity, cellular subpopulation, and their cross talks within the tumor microenvironment pertinent for clinical implications [8–10]. More recently, Juiz et al. [11] performed single-cell transcriptome analysis to determine the ITH particularly for PDAC epithelial compartment. They used 6 different classical patient samples taken from endoscopic ultrasound and fine needle aspiration. The single-cell transcriptomic analysis further differentiated samples into 4 main cell clusters, among which they found a cluster correspond to basal cell phenotype. They revealed from their analysis report that the basal-like cells were found to be highly aggressive and expected to widespread in the body. Furthermore, the stromal in the tumor microenvironment of PDAC consists of many cells like cancer-associated fibroblast (CAF) that found to have varied stromal heterogeneity and interacts with other cancer cells. Researchers showed that CAF in human PDAC displayed increased levels intratumor and intertumor heterogeneity. The subtyping for CAF-associated PDAC was classified based on the transcriptome analysis taken from various individual patients. The subtyping was profiled based on their gene expression and immunostaining markers that displayed phenotypic features of various proteins including vimentin, immune, and matrix-associated signatures and α-smooth muscle actin expression that are associated with the prognosis [12]. Thus, these classifications would reflect the development of therapies to target progressive cells in PDAC stroma.

### Disclosing mechanism of metastasis

The recurrent distant metastasis is the key cause for various cancer-related deaths. Consequently, a clear understanding of the mechanisms that are involved in developing metastasis promotes prevention of advanced metastatic cancer (Fig. 1). The irregular response to the adjuvant therapies is clinically heterogenous, causes metastasis due to the mutations majorly detected in four genes KRAS, SMAD4, TP53, and CDKN2A followed by their consecutive pathways [13]. A genome sequencing was performed for the clinical data collected and evidenced detection of 26 metastasis from 4 PC patients. They further determined a strong association between the primary tumor and metastasis that showed the same kind of mutations in the driver genes. Thus, this concept would strongly support a similar druggable target therapy to both primary tumor and metastatic tumor [14] which was also supported by the genomic investigation of Connor [15]. These cannot be evidenced through genomic studies alone and require transcriptomic analysis too [16, 17]. The analysis of transcriptome includes novel techniques for single-cell sequencing called single-cell RNA sequencing (ScRNA-seq) [18–20]. Thus, a whole genome sequence and RNA-sequencing analysis was performed for 319 PC samples that includes 95 from metastatic and 224 from primary site tumors. Additionally, 19 patients showed paired samples that is both primary and metastasis [15].

### Investigating mutated genes

Earlier researchers combined the capture system and next-generation sequencing analysis and determined the most frequently mutated genes including TP53, SMAD4, ARID1A, KRAS, CDKN2A, MLL3, and SF3B1 together added novel mutated genes: ATM and ARID2. Additionally, the novel gene signature ‘axon guidance’ genes (SLIT2 and ROBO2) are found to be highly associated with the PC pathogenesis [21]. Later, in support to this work, the whole genome sequence came into picture; wherein researchers determined 100 early-stage PC variation analysis that included gene mutations mainly in KRAS, SMAD4, TP53, CDKN2A along with Axon-guidance pathway [17] (Fig. 1). Basing on the chromosomal structural variations that are based on the DNA mechanism for PC pathogenesis, differentiated patients into four subtypes including stable, scattered, unstable, and locally arranged. For instance, in a clinical application among the 5 unstable patients who have undergone platinum-based therapy, 2 patients showed abnormalities for radiology treatment while the remaining 2 patients showed partial response. Thus, the response towards the platinum-based therapies can be taken as a predictive biomarker that showed a positive result in POLO trial who received Olaparib in PC patients with BRCA1/2 mutation [6]. Additionally, other than KRAS mutation, the somatic genetic modifications are also detected in alternative pathways including Ras/MAPK (missense mutation in GNAS gene). The mutations in BRAF and amplification of ERBB2 are the most frequently altered pathways detected in cancers [22]. Moreover, KRAS wild-type patients that showed recurrent fusion in genes NRG1 and RET are highly encouraged for targeted therapy for PC patients [23]. Similarly, KRAS wild-type patients with rearrangement in CTRC, NTRK showed that metastasis was found to be beneficial for tropomyosin receptor kinase inhibitor (TRK) is also encouraged for targeted therapy [24]. Accordingly, with The Cancer Genome Atlas, based on the miRNA expression, researchers determined 3 different miR clusters that were related to RNF43 mutations that are biologically and clinically relevant [25].

### Spotting circulating tumor cells

Precision medicine is another hope that includes liquid biopsy test for detecting circulating tumor cells (CTCs) and circulating tumor DNA (ctDNA) [26]. The CTCs are the malignant cells released from the primary site of PC lesion or metastatic site. These CTCs unexpectedly appear in the bloodstream of the cancer patient early before the metastatic lesions occur. Thus, CTCs plays a crucial role in predicting diseases progression and survival. Moreover, the single-cell RNA sequencing of CTCs promotes identifying dysregulated pathways and therapeutic targets (Fig. 1). The molecular analysis of CTCs was carried in bulk via mutational analysis, gene expression, and next-generation sequencing. These CTCs express epithelial cell adhesion molecule markers and a novel marker of fibroblast activation protein α. These makers are found to be distinct subpopulation and are used to navigate management of cancer [27]. Dimitrov et al. [28] isolated CTCs from the PC mouse models having liver and lung metastasis. They clustered the single-cell RNA sequencing as per their expressing profile including cell cycle molecules and extracellular matrix-associated genes taken from both primary and metastatic samples. The CTC transcriptomics identified survivin that is found upregulated in metastasis stage and a chief regulator protein of apoptosis and mitosis. Thus, single-cell RNA sequencing of CTCs promoted targeting of survivin inhibition by YM155 that is found to be beneficial for metastatic stage therapy in PC patients alone or in combination with chemo drugs, while the ctDNA was relevant as a biomarker for determining KRAS mutations and gemcitabine adjuvant chemotherapy in resected PC patients [29]. Thus, the genomic and transcriptomic classification study pictures the complicated heterogenous disease with varied subpopulation including tumoral, stromal, and immune cells and determines that cancers like PC is a complex of cancer cells. This could subsequently promote to categorize novel tumor and microenvironment-targeted therapies.

### History of transcriptome analysis for PC

Transcriptome is the fundamental study necessary for analysis of cancer based on the biological, prognostic, and clinical characteristics to define the essential molecular type of tumor. This analysis was started since early 2000s using DNA microarray technology. The initial transcriptomic analysis on PC is started by Collins et al. [30] using gene expression microarray analysis of 27 resected PC patients determined as 62-gene signature (PDAssigner gene set), which they differentiated into classical type (based on epithelial and adhesion related genes), exocrine (digestive enzyme genes), and quasi-mesenchymal type (mesenchymal-associated genes), and is illustrated clearly in Table 1. Later, Moffitt et al. [31] classified DNA microarray and RNA sequencing performed on virtual microdissection of 61 metastatic and 141 primary PC samples. They differentiated tumor subtypes as stroma and tumor related (Table 1). Bailey et al. [32] used whole genome sequencing and RNA-sequencing analysis to analyze 456 resected PC-differentiated tumor into squamous, immunogenic, pancreatic progenitor, and aberrantly differentiated endocrine exocrine (ADEX). Puleo et al. [33] in the year 2018 also analyzed 309 resected PC patient based on genome and transcriptome analysis and classified into classical and basal-like tumor subtypes. More recently, Chan et al. [34] also classified PC tumor subtypes into classical and basal like in accordance with the prognosis and putative markers. They analyzed about 330 cancer samples and performed whole genome sequencing. The hallmark for this classification is that the classical types showed upregulation of transcription factors including GATA4, GATA6, HNF4G, ONECUT2, and HNF1A with loss of SMAD4 but the limitation of this classification is that, the author fails to determine the correlation between the subtype and response to chemodrug regimen as they were able to analyze only few patients. However, basal-like subtype was found to be more benefitted from gemcitabine than mFOLFIRINOX. Further, the same research group determined that GATA6 expression could be a surrogate biomarker in COMPASS trial for chemo-resistance tumors. This specifies the chemotherapy regimen for malignancy as a new era of precision medicine [35, 36]. Thus, varied microenvironmental signatures can be differentiated based on varied clinical outcomes to subtype tumor. However, the complexity of the tumor environment cannot be determined by just RNA sequencing and therefore transcriptome of a single cell would be far more efficient to understand about the complexity of pathogenesis of PC.

**Table 1:** Classification of transcriptome

Transcriptome Study/Year	Properties	Classifications					References
Collisson et al. 2012		Classical Type	Quasi-Mesenchymal type	Exocrine type			[35]
	Biomarkers	GATA6 KRAS	Low or no expression of GATA6	ELA3A/ CFTR			
	Pharmacologically sensitive	Gemcitabine (low) Erlotinib (high)	Gemcitabine (high)				
	Prediction	Better survival	Worse overall survival				
Moffitt et al 2015		Stroma related		Tumor related			[31]
		Normal	Activated	Classical	Basal		
	Biomarkers	ACTA2, DES, VIM	ITGAM, CCL13, CCL18, FAP, SPARC, WNT2, MMP9, MMP11, WNT5a	GATA6	No expression of GATA6		
	Prediction	Good	Worst	Best	Worst		
	Pharmacologically sensitive			5-FU (high) Gemcitabine (low)	5-FU (low)		
Bailey et al		Squamous subtype	Pancreatic progenitor	ADEX	Immunogenic		[32]
	Biomarkers	GATA6 ↓and TP63↑	PDX1, FOXA2, FOXA3	MIST1, NR5A2	Immune cells (CD4^+^, CD8^+^), Check points (CTLA4, PD1)		
	Predictive	Poor	Good	Good	Good		
Puleo et al		Pure basal like	Pure/ immune classic	Stroma activated	Desmoplastic		[33]
	Biomarkers	GATA6↓, CDKN2A/ TP53	GATA ↑, CTLA4, immune cells (T & B cells)	α-SMA, FAP, SPARC	Stromal components		
	Predictive	Poor	Good	Average	Average		
Chang-seng-Yue et al		Classical	Basal	Hybrid			[34]
	Biomarkers	GATA4 ↑, GATA6↑	GATA6↓	GATA6 ↓↑			
	Pharmacologically sensitive	Gemcitabine(low) mFOLFIRINOX (high)	mFOLFIRINOX (low) gemcitabine (high)				
	Prediction	Good	Poor	Average			

### Other genomic analysis

The single-cell genomics is highly focused by the researchers at present to characterize the single tumor or stromal cells and analyze the transcriptome successively. The first single-cell RNA sequence was performed by Bernard et al. [20] in PC cells. They analyzed the microenvironment and transcriptional alteration correlated with the PC progression in the resected samples collected from 6 patients. The 6 patients taken were 2 complete PC, 2 low-grade, and 2 high-grade IPMNs. They almost sequenced 5403 single cells, however, only 3343 single cells are only considered and remaining are ignored for the reduced gene expression. They later detected 10 different clusters using the advanced t-distribution stochastic neighbor embedding method that showed unique stromal or epithelial cells with immune cells. The low-grade IPMNs from the epithelial cells were found with higher expression of oncogenes and cell cycle promoting gene while non-proliferating G_0_ phase is also detected in few other cells. However, a higher concentration of immune cells CD4^+^ and CD8^+^ cells are observed in both high and low-grade IPMNs with no levels of lymphocytes detected in PDAC lesions. The myeloid-derived suppressor cells (MDSCs) are highly detected in PDAC lesion (51%), whereas very rare in high-grade (3.5%) and low-grade (2.3%) IPMNs. The inflammatory cancer-associated fibroblasts (iCAFS) are highly detected in the clusters including VIM, CXCL12, IL6, and FAP. The myofibroblasts (myCAFs) responsible for endothelial growth factor secretions highly expressed α-SMA. Thus, this represents that there is a great shift of microenvironment cancer cell population over period with an increase in CAFs and reduction of T cells from low-grade IPMNs to high-grade IPMS and explicit PDAC lesions. This determines the tumor microenvironment and anti-cancer immune system results in the poor prognosis of cancer. In another study, PC cells were also found to secrete TGF-β and IL1 cytokines, whose signaling cascade activated myCAFs and inhibits iCAF. Alternatively, they also determined that CAFs activated by IL1 causes activation of JAK/STAT and are responsible for the activation of iCAF. These CAFs are interconvertible and rapidly shift from iCAF to myofibroblast with the inhibition of JAK pathway. Thus, there could be a novel possibility to direct drugs in the treatment of PC [37]. Accordingly, Dominguez et al. [18] also determined the presence of both subpopulation of CAFs including iCAF and myCAFs in PC as detected in the mouse model. Additionally, they also found that myCAFs cluster surrounds the cancer islet and promotes expression of leucine-rich repeat containing 15 (LRRC15). These proteins are found to enhance tumor progression in mouse model.

Furthermore, Moncada et al. [38] characterized spatial organization combining microarray and transcriptomics methods for gene expression using an array of spots. They also included multimodal interaction analysis to determine subpopulation of the primary pancreatic tumors. These include analysis of pancreatic ductal cells, dendritic cells and macrophages that maintains spatial restricted enrichments with other cells. Later, Elyada et al. [39] also performed single-cell sequencing for about 21.200 single cells taken from six human PDAC patients and 11.260 cells from 4 KPC PDAC mouse. They clustered about 15 cell clusters based on density-based clustering and determined immune cells including T/NK cells, B cells, dendritic cells, plasma cells, myeloid cells, and mast cells. Additionally, the clusters 1–3-4 are found to be malignant with high expression of hypoxia and inflammatory with the activation of KRAS and TP53. Another study including single-cell RNA sequencing was also performed to determine the immune cells expression in PC cells like T/NK cells and immune checkpoint receptors (CTLA4 and TIGIT) that have increased immune suppressive role in the tumor microenvironment [19]. Additionally, they also found expression of inflammatory fibroblast colocalization that are responsible for the expression of stress-response genes. Thus, this would be an advanced approach for mapping the single-cell RNA sequencing that defines inherent subpopulation of complex tumor tissue.

Peng J et al. [19] pictured a complete cellular ecosystem of PC to date in the single-cell transcriptomic atlas. They took 24 resected PC samples and 11 control pancreas and analyzed 41.986 and 15.544 single cells, respectively, and further differentiated them into 10 main clusters using principal component analysis. Among the clusters they have detected 2 ductal types: ductal type 1 and ductal type 2 that showed higher expression of ductal cell expression including MMP7, SOX9, KRT19, and TSPAN8. Wherein the ductal type 2 additionally showed higher expression of markers associated with poor prognosis including CECAM 1/5/6 and KRT19. Moreover, the control pancreatic cluster showed no such kind of expression and the ductal 2 is found to be composed of malignant cells. The seventh cluster was detected with markers CDK1, CCNB2, MK167, AURKA, PLK1, and CCNB1; they found inhibition of AURKA, CDK1, and PLK1 in vitro with drugs and can remarkably reduce survival of malignant cells survival.

T-cell receptor sequencing plays a crucial role in determining the efficacy of drug against the tumor cells. A combinational drug including pelareorep, pembrolizumab with a chemodrug showed increased efficiency with less toxicity as determined from the T-cell receptor sequencing. This sequencing done from the peripheral blood samples of PDAC patients showed new clones of T cells also with alterations in the immune gene expression found beneficial for patients clinically [40]. Pelareorep is an oncolytic reovirus drug used for solid tumor therapy which induces T-cell inflammation phenotypically in various cancers like PDAC. Earlier, single-cell RNA sequencing and T-cell receptor sequencing were done using syngeneic tumor models. They determined the alterations in infiltration of immune cells in distant before and after radiofrequency-ablated (RFA) tumor cells. They identified distinct clusters from their single-cell RNA sequencing that include 6 clusters for lymphoid, 5 for monocytes, one for neutrophils, and 3 for dendritic cells. The RFA-treated PC cells showed decreased proportions of immunosuppressive cells like tumor-associated neutrophils, macrophages, and regulatory T cells with an increased concentration of T cells in non-RFA PC cells. The biological progression of tumor-infiltrating CD8^+^ T cells and monocytes was also transcriptionally profiled based on the pseudo-time analysis. Additionally, the PC cells from the clusters also showed increased upregulation of immune checkpoints: LAG3 and PD1. Thus, this approach of combining RFA with immune cells check point suggests a novel effective therapy for PC cells [41]. Furthermore, various advances like patient-derived organoids for PC patients are highly advisable for characterizing genomic and transcriptomic studies for primary PC and help to establish therapeutic strategies based on the sensitivity and resistance developed by the cells [42].

### Novel developments in single-cell sequencing techniques

The researchers are now moving on to advanced single-cell sequencing techniques in order to lower the detection cost on the molecular mechanism at the level of single-cell study. Table 2 illustrates current advanced single-cell sequencing techniques. Furthermore, the cost reduction was also found beneficial with the combination of single-cell sequencing to other technologies. For instance, CRISPR screening is a combination with scRNA sequencing that enables the functional analysis of heterogenous cell population and facilitates study of inter-relationship between genes and regulatory elements [43, 44]. Combining sNuc-seq with microfluidic technology, this combinational method promotes introducing single-cell nuclear RNA sequencing to develop a highly efficient and sensitive classification [45]. These advanced technical processes facilitate construction of complete map for single cells and could be powerful tools to diagnose and treat cancers.

**Table 2:** Advanced single-cell sequencing techniques

Single-cell sequencing technique	Function	Abstract	References
Single-cell combinatorial marker sequencing technique (SCI-seq)	Detects somatic cell variations and constructs thousands of single-cell libraries	SCI-seq analysis done to generate thousands of single-cell libraries for variant detection of somatic copy number within in PC. The libraries constructed from 16,698 single cells taken from primate frontal cortex tissue and 2 human adenocarcinomas	[46]
Single-cell whole genome amplification method (WGA)	Can efficiently detect mutations in multiple diseases	KRAS mutations in CTCs were detected with a rate of about 27.7% from samples 11 of 12 PC patients. Moreover, KRAS mutations were found in WBC sequenced cells	[47]
Topographic single-cell sequencing (TSCS)	Describes spatial characteristics invasion and metastasis of tumor cells	KRAS mutations in CTCs were detected with a rate of about 27.7% from samples 11 of 12 PC patients. Moreover, KRAS mutations were found in WBC sequenced cells	[48]
Single-cell multiple sequencing technique (scCOOL-seq)	Analysis of single-cell chromatin state, DNA methylation	Measures genomic number profile of a single tumor cell while preserving the spatial context in tissue sections taken from both ductal adenocarcinoma in situ and invasive ductal carcinoma of 10 synchronous patients. Additionally, a direct lineage was determined in between invasive and in situ tumor cells that shows aberrations evolved and mutations present within the ducts prior to invasion	[49]

## Conclusion

PC remains the principal cause for mortality globally, whereas the tumor heterogeneity is the hallmark for progression of PC. Additionally, it also indulges to respond variedly to therapeutic regimens. The single-cell sequencing technology is a powerful tool to detect rare cancer stem cells, epigenetic alterations, ITH and develop personalized therapeutic strategies. Furthermore, mapping each and every individual cell promotes understanding the relationship between cells, their physiological and pathological mechanism to find novel diagnostic biomarkers to be targeted by the therapeutic drugs. Future more advances were made in single-cell technology with the launching of Human Cell Atlas (HCA) project and Human Biomolecular Atlas Program (HuBMAP) for dissecting each and every single tumor cell using genomic tools. The new genome sequencing is significant to discover new drugs against newly detected mutations. Thus, the drugs would be efficiently targeted to address the genomic, transcriptomic, and genetic heterogeneity. However, single-cell sequencing technology is limited due to its extreme cost, experimental time, and not so popular in use. Advances in these areas could promote application of single-cell technology in the field of cancer for diagnosis, therapy, and the survival of the patients.

## Abbreviations

ADEX: Aberrantly differentiated endocrine-exocrine
CAF: Cancer-associated fibroblast
CSC: Cancer stem cells
CTC: Circulating tumor cells
ctDNA: Circulating tumor DNA
FCA: Flow cytometry analysis
HCA: Human cell atlas project
HuBMA: Human biomolecular atlas program
iCAFS: Inflammatory cancer-associated fibroblasts
IPMN: Intraductal papillary mucosal neoplasms
ITH: Intratumoral heterogeneity
LCM: Laser capture microdissection
LRRC15: Leucine-rich repeat containing 15
MDSC: Myeloid-derived suppressor cells
MSI: Microsatellite instability
myCAFS: Myofibroblasts
PARP: Poly (ADP-ribose)polymerase
PC: Pancreatic cancer
PDAC: Pancreatic ductal adenocarcinoma
RFA: Radiofrequency ablated
SCI0seq: Single-cell combinatorial marker sequencing
scRNA-seq: Single-cell RNA sequencing
TRK: Tropomyosin receptor kinase inhibitor
TSCS: Topographic single-cell sequencing
WGA: Whole genome amplification
